# Post-Operative Low Cerebrospinal Fluid Pressure Headache in Giant Tarlov Cysts

**DOI:** 10.7759/cureus.27045

**Published:** 2022-07-20

**Authors:** Sakshi Kadian, Swathi Mallikarjuna, Vikram Chandra, Sanjay Agrawal

**Affiliations:** 1 Anaesthesiology and Critical Care, All India Institute of Medical Sciences, Rishikesh, IND

**Keywords:** marsupialisation, headache, cerebrospinal fluid leak, laminectomy, giant tarlov cyst

## Abstract

Giant Tarlov cysts are described as fluid-filled sacs located in the spine. They are mostly found in the sacral region, but are usually asymptomatic. The symptomatic Tarlov cysts are planned for surgical treatment in the form of laminectomy and marsupilisation of cysts. These surgical procedures can have complications like cerebrospinal fluid (CSF) leak, bacterial meningitis, and radiculopathic pain. We report a case of a 30-year-old male who presented with complaints of pain in his left leg for one and half years, urinary incontinence off and on for six months, and scrotal pain for two months. He was diagnosed with two giant Tarlov cysts on contrast-enhanced magnetic resonance imaging and planned for surgery. Intraoperatively, approximately 1000 ml of CSF was drained. The patient complained of severe headache in the immediate postoperative period, which was confirmed to be a low-CSF pressure headache. Prompt diagnosis and management with collaborative teamwork of neuroanesthetists and neurosurgeons helped treat the patient and prevent long-term morbidity.

## Introduction

Tarlov cysts are fluid-filled sacs occurring in the spine, commonly in the sacral region. Tarlov first described them as incidental autopsy findings. Tarlov cyst have a reported incidence of 1%-5% [1]. They are usually asymptomatic, and large symptomatic cysts are rare. Surgical treatment of symptomatic cysts includes laminectomy and drainage of cyst along with marsupialisation or occlusion of cyst cavity with fibrin glue. Surgical complications include cerebrospinal fluid (CSF) leak, bacterial meningitis, new-onset radiculopathic pain, worsening existing pain, and bladder or bowel-related complications. Low-CSF headaches are caused by low CSF volume or pressure, which can be spontaneous or iatrogenic [2]. 

We report a case of low-CSF pressure headache as a complication of S1-S2 laminectomy and marsupialisation of giant Tarlov cysts in the sacral region.

## Case presentation

A 30-year-old male presented to the neurosurgical outpatient unit with complaints of pain in the left leg radiating to the sole for one and a half years, urinary incontinence off and on for six months, and scrotal pain for two months. There was no significant history or known comorbidities. On examination, grossly dilated, tortuous veins were seen in bilateral lower limbs suggestive of varicose veins. Sensory and motor examinations were normal, and other systems examinations were unremarkable.

Investigation

**Figure 1:**
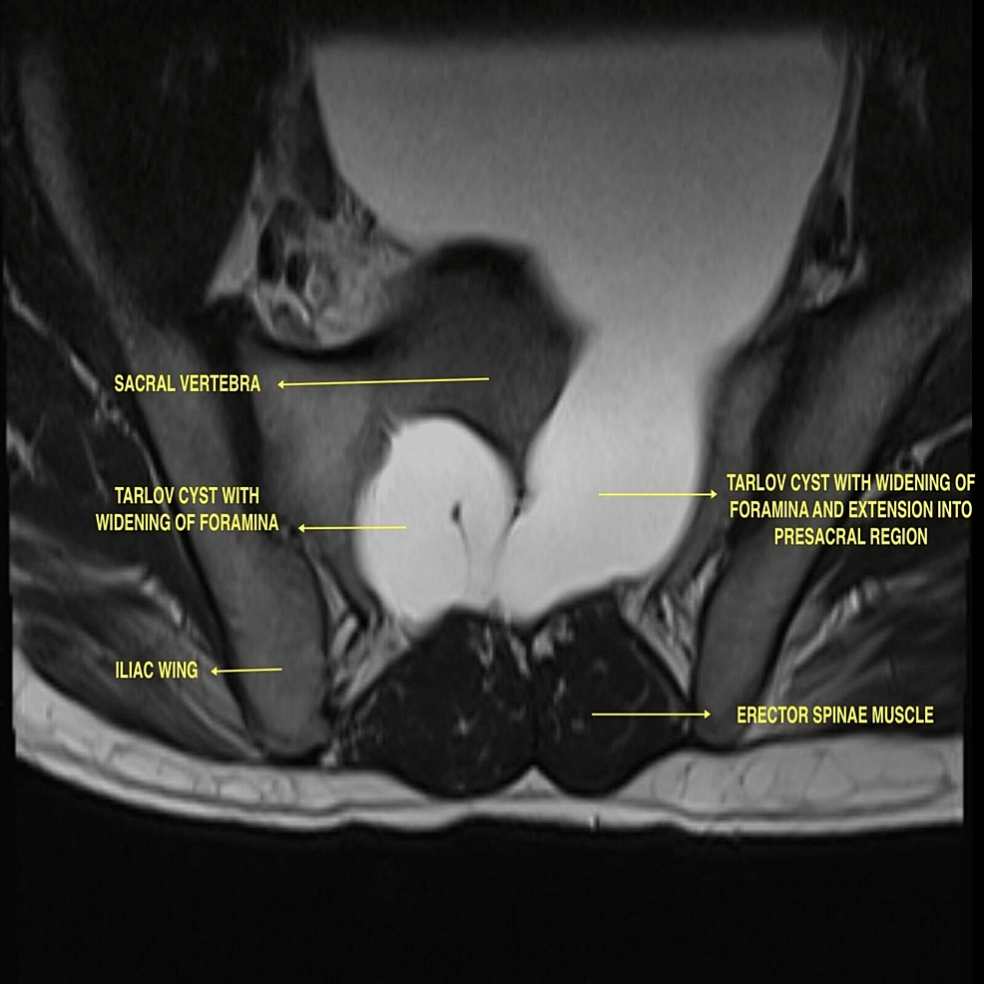
T2 MRI transverse axial image depicting giant Tarlov cysts

**Figure 2:**
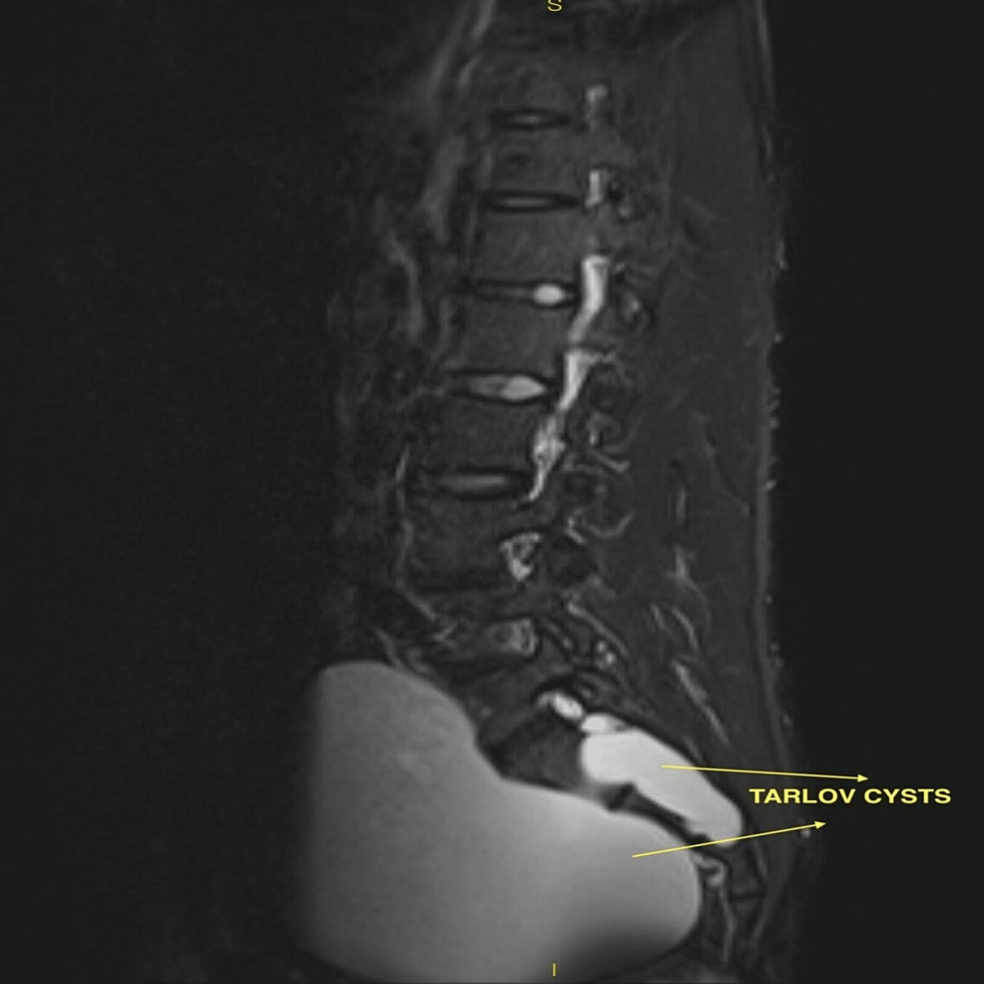
T2 MRI sagittal image showing giant Tarlov cysts

Ultrasound venous Doppler of the left lower limb revealed multiple incompetent perforators with left incompetent saphenofemoral valves, and the diagnosis of left varicose veins was confirmed. Abdominopelvic ultrasound revealed an anechoic cystic lesion of size 12×11×11.5 cm in the pelvic region in the presacral area, compressing the urinary bladder. Contrast-enhanced computed tomography of the whole abdomen revealed two low-density non-enhancing presacral space-occupying lesions arising from the sacral canal and bilateral neural foramina at the S1-S2 level. Contrast-enhanced magnetic resonance imaging of the lumbosacral and whole spine revealed two well-defined cystic lesions measuring 2.8×5.3×3.3 cm on the right side and 12.8×12.2×13.2 cm on the left side with mass effect on the urinary bladder and extending anteriorly in the presacral space and traversing bilateral S2 neural foramina (Figures 1-2).

A provisional diagnosis of Tarlov cysts was made, and the patient was planned for laminectomy and marsupialisation of cysts in the prone position under general anaesthesia. Preoperative blood investigations were routine, and the pre-anaesthetic check-up was insignificant.

Treatment

Standard anaesthesia technique as per institute protocol was employed for general anaesthesia. Induction of anaesthesia was achieved with injection (inj) fentanyl two mcg/kg intravenously (IV), propofol two mg/kg IV, and muscle relaxation was achieved with inj. vecuronium 0.1 mg/kg. The trachea was intubated with an appropriate-sized cuffed endotracheal tube, and anaesthesia was maintained with 40% oxygen in the air and sevoflurane at 1.0 minimum alveolar concentration (MAC). The patient was positioned prone on the Allen frame, and cushioning of all pressure points was done. Laminectomy was performed at the S1-S2 level, and the sacs of cysts were identified, extending into the pelvis. During cyst excision, the cyst ruptured with a loss of approximately 1000 ml of CSF, which was replaced with an additional transfusion of Ringer’s lactate. The rest of the course of surgery was uneventful. Bilateral erector spinae plane block was given with 20 ml of 0.25% bupivacaine at the level of L4 for postoperative pain relief under ultrasound guidance. The patient was extubated on the operation table.

On arrival at the post anaesthesia care unit (PACU), the patient complained of a severe headache. It was sudden onset, progressive, occipital in location, and throbbing in nature. Queckenstedt's test demonstrated a low-pressure headache, managed per post-dural puncture headache protocol. He was shifted to the ICU and was advised complete bed rest with head straight for the next 48 hours, and given IV fluids, and NSAID for pain relief. The headache persisted for 4-5 hours, and the patient was advised caffeine tablets following which his symptom improved.

Outcome and follow-up

The patient was relieved of headache on the second postoperative day and discharged after one week. His symptoms of tingling in the legs, back pain, and urinary incontinence were relieved. On follow-up, his signs were all relieved, and he was satisfied.

## Discussion

Tarlov cysts, first described in 1938, are categorised as spinal arachnoid cysts. They are even described by the National Organisation of Rare Diseases (NORD), the National Institutes of Health, and the Genetic and Rare Diseases Information Center (GARD). They may be extradural or intradural in location and may or may not be accompanied by nerve roots involvement. Spinal extradural cysts account for approximately 1% of lesions [3]. Nabros has classified spinal arachnoid cysts according to location and involvement as extradural cysts without and with nerve root involvement are Type I and Type II cysts, respectively [4]. All intradural cysts are Type III spinal arachnoid cysts.

This case had an extradural cyst involving nerve roots at the S1-S2 vertebral level (Type II cysts). Such cysts are commonly seen in females in the age group of 20-50 years. They most often arise from nerve roots of S2-S3 of the sacral spine [5]. The aetiology remains unclear and may be congenital or acquired, secondary to trauma, infection, inflammation, or iatrogenic causes. Bone erosion and expansion of cysts are commonly seen in gaint Tarlov cysts. The clinical presentation includes back pain, radiculopathy, coccydynia, dyspareunia, and bowel and bladder incontinence. Giant Tarlov cysts are reported to have a free flow of CSF between the cyst body and subarachnoid space [6].

Low-CSF pressure headaches are secondary to CSF volume or pressure. It can be spontaneous or provoked, such as inadvertent dural puncture in spinal anaesthesia, after a lumbar puncture, or neurosurgical procedures. Postural headache is the hallmark of intracranial hypotension; the headache peaks within minutes of being upright and gets relieved on lying down. The headache is throbbing in quality, frontal or occipital in location, and often generalised with neck pain. Other associated symptoms include nausea, vomiting, diplopia, blurred vision, and tinnitus [2]. Tarlov cysts that rupture account for most incidences of spontaneous intracranial hypotension. Usually, it is a benign entity but can result in significant neurological deficits, obtundation, and death [7]. They are typically managed with adequate hydration, bed rest, NSAIDs, and caffeine. In refractory cases, an epidural blood patch or injection of fibrin sealant may be needed. The marsupialisation of giant Tarlov cysts results in high volume drainage and severe symptomatic intracranial hypotension, requiring surgical intervention to repair the leak [8].

In our case, the giant Tarlov cysts presented with varicocele and left leg varicose veins, which are uncommon presentations. The possible explanation for this presentation could be the increased intra-abdominal pressure owing to these large cysts. However, the typical symptoms of radiculopathy and bladder incontinence were also present. The fasting hours were kept as per the American Society of Anesthesia (ASA). After obtaining the patient’s consent, the patient was taken to the operation theatre for general anaesthesia. Intraoperatively, intraarterial blood pressure and pulse pressure variation monitored the patient's volume status. Fluid deficits were replenished with IV fluid, with 0.9% normal saline as the fluid of choice. During marsupialisation of the cyst, the volume of CSF lost was adequately replaced with maintenance fluids, maintaining the intravascular volume and blood pressure. There was no episode of hemodynamic instability. The postoperative complication of low-pressure headache was diagnosed immediately and managed with fluids, bed rest, and NSAIDs. A caffeine tablet was given once oral feeds were started. The patient was relieved of symptoms due to the early intervention and joint efforts of neuroanaesthesiologists and neurosurgeons.

## Conclusions

The drainage of a large amount of CSF volume during marsupialisation can cause postoperative morbidity in the form of low-CSF pressure headaches. It should be immediately managed to prevent patient discomfort and avoid neurological deficits.
